# Maternal and Child Health Services Utilization During COVID-19 in Eastern Ethiopia

**DOI:** 10.3389/ijph.2024.1606626

**Published:** 2024-05-22

**Authors:** Bereket Tefera Tilahun, Zerihun Tariku, Mesfin Kebede Alemu, Tafese Dejene, Legesse Abera Natae, Hussen Mohammed, Daniel Tadesse Assegid, Mickiale Hailu Tekle

**Affiliations:** ^1^ Department of Public Health, College of Medicine and Health Science, Dire Dawa University, Dire Dawa, Ethiopia; ^2^ School of Medicine, College of Medicine and Health Sciences, Dire Dawa University, Dire Dawa, Ethiopia; ^3^ Department of Midwifery, College of Medicine and Health Science, Dire Dawa University, Dire Dawa, Ethiopia

**Keywords:** maternal health, child health, MCH service utilization, COVID-19, Eastern Ethiopia

## Abstract

**Objectives:** This study aimed to describe the impact of COVID-19 on maternal and child health service utilization in the Eastern part of Ethiopia.

**Methods:** Comparative analysis was used to examine 2 years of maternal and child health service utilization. Data were extracted from client registers. A traditional Expert Modeler and one-way Analysis of Variance were used to compare service utilization before and during the COVID-19 pandemic.

**Results:** A total of 34,576 client records were reviewed, of which 17,100 (49.5%) and 17,476 (50.5%) had visited the MCH service before and during the COVID-19 pandemic, respectively. The total client visit has shown a 2% percentage point increase. However, postnatal care and child immunization services showed a decrease. Moreover, there was a significant difference between service visits before and during COVID-19 (f = 4.6, *p* < 0.04).

**Conclusion:** Mothers and children have missed or unattended facility appointments due to protective impositions or fear of getting infected with COVID-19, which might suggest a higher proportion of MCH issues were not addressed during the pandemic. The health system should therefore improve its resilience and strengthen its access at the lowest health care inlets.

## Introduction

Maternal and child health should be a positive experience at every stage of the continuum, during pregnancy, delivery, and the postpartum period, in addition to the newborns childhood [1]. Family planning, prenatal care, childbirth, and postnatal care services are all parts of maternal health care services. Infant feeding practices, anthropometry-measured nutritional status, and childhood immunization history are important indicators of child health [2]. Furthermore, protecting and improving the health of children is of fundamental importance and a crucial component of maternal and child health [1]. A large proportion of maternal and neonatal deaths occur in the first 48 h after birth. Thus, prompt postnatal care (PNC) for both the mother and the child is important to treat any complications arising from childbirth and to provide the mother with important information on how to care for herself and her child [2].

Numerous studies conducted worldwide during the COVID-19 pandemic have shown that there has been a significant decline in the use of healthcare services, as clients have canceled appointments and showed a decreased desire to seek medical attention [4–6]. Timothy Roberton and colleagues have modeled the impact of COVID-19 on 118 low- and middle-income countries. They have also presented startling new data regarding the possibility of an increase in maternal and child mortality in the event that COVID-19 causes disruptions to key health services. Drawing on past outbreaks of the Ebola virus disease and severe acute respiratory syndrome (SARS) outbreaks, the authors predict a catastrophic rise in maternal and child mortality rates as a result of declining coverage of regular health services [4]. Conversely, the majority of services provided by medical facilities have been compromised by the presence of COVID-19. According to the findings of the most recent key informant pulse survey, there have been disruptions of crucial health services in almost every responding country, with lower-income nations experiencing this phenomenon more frequently than higher-income ones. Multiple services have been impacted, such as critical mental healthcare, reproductive, maternal, newborn, child, and adolescent healthcare; non-communicable diseases; and nutrition services [5, 6]. Hard-won progress in improving the health of women, children, and adolescents was at risk due to the COVID-19 pandemic. The virus itself poses less of a risk to women and children, but its knock-on consequences, like reduced access to resources for sexual and reproductive health and vaccinations, are expected to cause significant harm [7, 8].

The COVID-19 pandemic threatens to overwhelm health systems and undo progress made in maternal, neonatal, and child health (MNCH) outcomes. It necessitates a change in resources, including budget, health workforce, facilities, logistics, and supplies. Health-seeking behaviors have also been impacted by the COVID-19 pandemic. Families and communities with limited transportation alternatives and concerns about contracting an infection in medical facilities were less likely to utilize basic health services [10–12]. Ethiopia has one of the highest maternal mortality rates worldwide. A woman’s annual risk of dying from childbirth-related causes is one in fifty-two. In Ethiopia, 120,000 neonates and over 257,000 children under the age of five die annually. In Ethiopia, neonatal fatalities account for 40% of under-five deaths and over 60% of infant mortality [9]. Based on current data, it is anticipated that the presence of COVID-19 will result in a multiplication of this burden.

In this study, we were interested in analyzing the impact of COVID-19 on the utilization of maternal and child health services in Dire Dawa, Eastern Ethiopia. A comparative analysis was conducted comparing the 12-month period before COVID-19 and the 12 months during which mothers and children accessed MCH services following the declaration of the outbreak in Ethiopia on 12 March 2020. The data sources for this investigation were the MCH client registry and the follow-up logbooks of the public health facilities involved.

### Impacts of COVID-19 on Maternal and Child Health Service Utilization

The impact of COVID-19 on 155 nations was evaluated by the World Health Organization (WHO) over a 3-week period in May 2020. The results showed that several countries experienced partial or total disruption of health services [6]. Similarly, a global assessment by the Global Financing Facility of 63,000 health institutions revealed that COVID-19 disrupted important health and mental health services in low-income countries. Significant variations in the provision of outpatient care, childhood vaccinations, care for expectant mothers and newborns, and skilled deliveries were observed in multiple nations [8].

According to a study conducted in Bangladesh, Nigeria, and South Africa to evaluate the impact of MNCH service utilization, there was a decline in ANC attendance in April and May 2020 compared to the same period in 2019. Declines in attendance were also observed for family planning and child immunization services. In particular, Bangladesh has seen a decline in vaginal births and C-sections, while South Africa and Nigeria have seen mixed results. Factors such as the lack of personal protective equipment for health workers and the enforcement of lockdowns and similar procedures have been implicated [10]. According to a retrospective study carried out in Nepal, prenatal care and the use of conventional delivery services decreased at the start of the COVID-19 lockdown but increased over time. There was a mixed trend in the use of permanent family planning methods and cesarean sections, while the usage of vaccination services and temporary family planning methods increased over time [11]. A 7.2% increase in high-risk pregnancies was linked to a decrease in institutional deliveries, according to a prospective observational study carried out in India. Similarly, poor antenatal care follow-up was reported in one-third of pregnant women [12]. Another comparative analysis conducted in India found an overall decline of 2.26% in all maternal and child health services, with the greatest impact on prenatal care services, which declined by 22.9%, and vaccination services, which declined by 20% [13].

A multi-country, multi-site study conducted in eight sub-Saharan countries found that there was a disruption in the use of health services for at least 1 month during the COVID-19 pandemic in all countries investigated, although the extent and duration varied from country to country. The most impacted service was the child immunization program. Although there was a decline in the use of maternal health services, not everyone experienced this. Nonetheless, in certain nations, there were noticeable reductions in hospital deliveries and both prenatal and postnatal services [14]. In the first 2 months of COVID-19, there was no difference in the number of people attending reproductive, maternal, newborn, child, and adolescent health (RMNCAH) service points, hospital births, family planning visits, post-abortion care, and pentavalent 1 immunization, according to a 4-month comparative study conducted in Kenya. However, there were noticeable increases in the rate of cesarean sections, the uptake of injectable family planning methods, and adolescent pregnancies and family planning use, and the uptake of injectable family planning methods [15]. A study conducted in Rwanda took MCH data from 30 districts between March and April 2019 and compared it to the same months in 2020 to see if seasonal fluctuations affected service utilization in light of COVID-19. The findings indicated a remarkable decline in prenatal care, institutional deliveries, postnatal care, and childhood immunizations [16]. In an analysis conducted in the KwaZulu-Natal district of South Africa to evaluate the effects of COVID-19 on routine child health services, a decrease in immunization services was noted early in the pandemic. During the COVID-19 era, vitamin A supplementation remained low. The study also found that there was a noticeable disruption in several service indicators related to child wellbeing, service delivery, and access to services [17]. A reduction in hospital deliveries, c-sections, family planning consultations, and child vaccinations was also confirmed by a mixed-methods comparative study conducted in Mozambique [18].

Similarly, the COVID-19 pandemic resulted in the disruption of MCH services in different parts of Ethiopia. According to a qualitative study, the use of antenatal care services was altered by COVID-19 [19]. Another 4-month comparative research conducted in Ethiopian public health facilities revealed a decrease in the mean use of family planning, prenatal care, and newborn vaccinations. At the same time, during the same period, there was an increase in institutional stillbirths, teenage pregnancies, abortions, and neonatal deaths [20]. An analogous study in Dessie Town, Ethiopia, revealed a decline in the use of prenatal, birth, and postnatal care attendant services [21]. In order to assess the provision of essential health and nutrition services in four different regions of Ethiopia—Amhara, Oromia, the Southern Nations and Nationalities Region, and Tigray—a mixed-methods comparison study was conducted. Reductions in service delivery and utilization were reported by key informants from healthcare facilities. A review of the patterns in maternal nutrition and health service use from March to July 2019 and 2020 revealed a slight decline in antenatal care and facility deliveries. On the other hand, an observable decline was noted in the provision of vitamin A supplements and feeding services for infants and young children. However, there was little variation in other child health services [22]. A study carried out by the JSI L10K project on 96 health facilities in Ethiopia revealed that in April 2020, compared to the average performance over the preceding 8 months, there was a 12% decline in first antenatal attendance and a 35% fall in under-five pneumonia treatment [23].

Therefore, this study can help us learn how much COVID-19 has impacted the use of health services in the study area by examining the pattern of MCH service utilization. In addition to the already fragile health system in low-income nations like Ethiopia, a severe pandemic such as COVID-19 could jeopardize maternal and child health service utilization and its outcomes, in addition to the likelihood of covert maternal and child mortality.

This research identifies the service unit that has been most affected by the COVID-19 pandemic. Additionally, it can provide program administrators with information about the type of service being targeted, assist in the strategic allocation of labor and resources to improve client flow and service coverage, intensify focused health communication, and address potentially pressing issues to revive MCH service utilization.

## Methods

The study was conducted in Dire Dawa, in the eastern part of Ethiopia, in selected public health facilities and hospitals. The Dire Dawa Administration is a large city, and one of the two federal-level city administrations, the other being Addis Ababa, the capital of Ethiopia. Dire Dawa serves as an international business corridor connecting the eastern region of Ethiopia to neighboring countries. It facilitates the import and export of national commodities through land, air, and sea transportation. Additionally, Dire Dawa is a crossroads for the regional states of Harari, Somalia, and Oromia. Most importantly, the Eastern Isolation and Quarantine Center was established in Dire Dawa for migrants arriving in Ethiopia as a result of the COVID-19 pandemic. Dire Dawa became the epicenter of the COVID-19 pandemic due to all of the above-described circumstances, which greatly impacted the healthcare system [3, 4].

The study area has two public hospitals and 16 public health centers. According to the Dire Dawa Administration, the projected population in 2022 was 550,642, of which 68% were urban inhabitants. For this study, simple random sampling was employed to select five public health centers and one public referral hospital. Data were collected from the first of February to 9 March 2021. The difference in MCH service utilization between two 12-month periods—before and during the COVID-19 pandemic—was investigated using comparative analysis. The declaration of the presence of COVID-19 in Ethiopia on 12 March 2020, was used as the interruption point. For comparison, we measured the 12 months before and after this midpoint.

Records of mothers and children who visited family planning, prenatal care, birth, postnatal care, and child immunization services throughout the study period were extracted from the register logs. Kobo toolbox, a data collection, management and visualization platform, was used to create the data collection instrument. Accordingly, a structured checklist that matches the national MCH service utilization registry was constructed to gather the necessary data. Facility and service entry point details, period selection, client age at service entry, and service use characteristics were all captured by the data collection tool.

After receiving ethical approval from the Dire Dawa University Research Ethics Review Committee to conduct the study (Ethical Approval Protocol Number: DDU-ERC-2020-012), we commenced the study process.

The data collection instrument was rigorously tested and made available to the data collectors. For the purpose of data collection, trained midwives were assigned to the selected facilities. Once approval was obtained from the regional health bureau and selected facility directors, the data collection began. All safety precautions for COVID-19 prevention were taken into account while monitoring and interacting with the research team.

Following data clearing in MS Excel, the collected data were exported to SPSS V26 for analysis. The frequencies, averages, and percentages of descriptive findings are presented. Service utilization patterns and disruption effects were examined in the data using a traditional expert modeler. Service utilization was plotted and fit lines were compared at a 95% confidence interval, with stationary R square used to reflect the level of prediction. The total number of clients who visited the facilities as well as specific service utilization patterns were tracked by sequential charting. To compare the impact of COVID-19 on the use of maternal and child health services, percentage points were calculated. A one-way ANOVA was carried out to determine whether patient attendance patterns differed between the two time periods.

## Results

Records from 10 March 2019 to 9 March 2021 were taken from the Maternal and Child Health Service Units of the selected facilities, with 12 March 2020 considered the interruption point. A total of 34,576 pieces of data were extracted from six facilities. As a result, 49.5% (17,100) of the MCH service visits were made prior to the announcement of the COVID-19 pandemic in Ethiopia (10 March 2019 to 11 March 2020), while 50.5% (17,476) of the visits occurred during the pandemic (12 March 2020 to 9 March 2021) (Table 1).

**TABLE 1:** Client facility visits for maternal and child health services utilization during COVID-19, at selected health facilities (Eastern Ethiopia, 2021).

Facility	Before COVID-19 n (%)	During COVID-19 n (%)	Totals n (%)
Addis Ketema HC	1,794 (49.4%)	1,836 (50.6%)	3,630 (10.5%)
Dechatu HC	2,065 (47.7%)	2,264 (52.3%)	4,329 (12.5%)
Gende Gerada HC	2,964 (49.6%)	3,013 (50.4%)	5,977 (17.3%)
Gende Kore HC	2,518 (48.9%)	2,628 (51.1%)	5,146 (14.9%)
Jelo Belina HC	1,665 (52.5%)	1,504 (47.5%)	3,169 (9.2%)
Dil Chora Hospital	6,094 (49.4%)	6,231 (50.6%)	12,325 (35.6%)
Mean Service Visit	**1425.0**	**1456.3**	
Totals	**17,100 (49.5%)**	**17,476 (50.5%)**	**34,576 (100%)**

Mean service visits represent: Average number of clients visited the facility for MCH service during the period, before Covid-19 1425, and during Covid-19 1456.3. Totals represent: Total number of clients visited the facility for MCH services during the period, before Covid-19 17,100 (49.5%), during Covid-19 17,476 (50.5%), and total number of clients visited the facilities during the two periods were 34,576 (100%).

During the study periods, the highest proportion of MCH service visits were made to Child Immunization at 25.1% (8,663) and Family Planning Services at 24.7% (8,552), while the lowest proportion of visits were made to Postnatal Care Services at 8.1% (2,817) (Table 2).

**TABLE 2:** Client service visits for maternal and child health services utilization during COVID-19, at selected health facilities (Eastern Ethiopia, 2021).

MCH services	Before COVID-19 n (%)	During COVID-19 n (%)	Totals n (%)
Postnatal Care	1,657 (58.8%)	1,160 (41.2%)	2,817 (8.1%)
Family Planning	4,152 (48.6%)	4,400 (51.4%%)	8,552 (24.7%)
Delivery	3,053 (44.2%)	3,861 (55.8%)	6,914 (20%)
Child Immunization	4,716 (54.4%)	3,947 (45.6%)	8,663 (25.1%)
Antenatal Care	3,522 (46.2%)	4,108 (53.8%)	7,630 (22.1)
Totals	**17,100 (49.5%)**	**17,476 (50.5%)**	**34,576 (100%)**

Totals represent: Total number of clients visited all MCH services during the period, before Covid-19 17,100 (49.5%), during Covid-19 17,476 (50.5%), and total number of clients visited all MCH service units in the study facilities during the two periods were 34,576 (100%).

### Maternal and Child Health Service Utilization:

**FIGURE 1:**
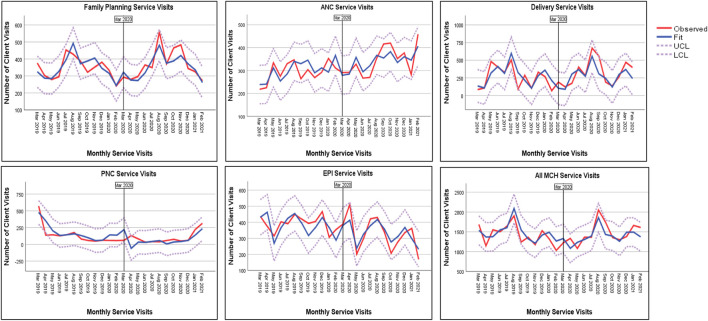
Utilization of maternal and child health services during COVID-19 at selected health facilities (Eastern Ethiopia, 2021).

Time series data for Years and Months with a 12-month periodicity were first defined using the Date and Time wizard. In order to characterize the client’s 12-month visit, we fit the data to the traditional expert modeler. It was observed that there was a decrease in the overall use of maternal and child health services in the research area since the declaration of the COVID-19 pandemic in Ethiopia. After 6 months, from August to September 2020, the service utilization had improved, but in October of the same year, there was a substantial decrease. Here, the fitted model has explained 82.4% (*p* < 0.01) of the cumulative service utilization. Family planning service utilization did not show a significant difference before and during the time of the COVID-19 pandemic (*R* ^2^ = 87.3%, *p* < 0.26). ANC service utilization has shown a steadily increasing pattern during the same period (*R* ^2^ = 84.3%, *p* < 0.02). The general pattern of delivery service utilization was irregular. We observed a decreasing trend since September 2019, where a steady rise was observed from June 2020 and reached its peak in September 2020 followed by a sharp fall in December 2020 (*R* ^2^ = 82.4%, *p* < 0.01). There was no apparent shift in the pattern of postnatal care service utilization over the course of the study, with the monthly client visits below 250 (*R* ^2^ = 78.3%, *p* < 0.19). Child immunization services have shown a steep decline following the announcement of the COVID-19 pandemic, peaking in the months of April and May 2020. Since then, there has been a mixed pattern of 200–400 client visits per month (*R* ^2^ = 87.1%, *p* < 0.01) (Table 3, Figure 1).

**TABLE 3:** Trend analysis model statistics for maternal and child health services utilization during COVID-19 at selected health facilities (Eastern Ethiopia, 2021).

Service departments	*R* ^2^	df	P
Family Planning Service	0.873	16	0.257
Antenatal Care Service	0.843	15	0.020^a^
Delivery Service	0.824	16	0.006^a^
Postnatal Care Service	0.783	16	0.198
Child Immunization Service	0.871	15	0.015^a^
Maternal and Child Health Service (Total Visits)	0.824	16	0.017^a^

^a^ Statistically significant differences were observed during the comparison periods of service utilization.

An analysis of the data was later conducted to determine how COVID-19 affected the percentage point and percentage shift in MCH service utilization. There was a 2% difference in the total number of patient visits in the study area before and after the announcement of the COVID-19 pandemic. Similarly, there was a 6% percentage point increase in the use of family planning services, a 26% percentage point increase in the use of delivery services, and a 17% percentage point increase in the use of antenatal care services. However, there were decreases of 30% and 16% percentage points in the use of postnatal care and child immunization services, respectively (Table 4).

**TABLE 4:** Impact of COVID-19 on maternal and child services utilization during COVID-19 at selected health facilities (Eastern Ethiopia, 2021).

Services	Client visit means _0_	Client visit means_1_	% point mean _1_ - mean _2_	% (PP/Means _0_) ×100 (%)
Postnatal Care Service	138.1	96.7	−41.4	−30
Family Planning Service	346	366.7	20.7	6
Delivery Service	254.4	321.8	67.4	26
Vaccination Service	393	328.9	−64.1	−16
Antenatal Care Service	293.5	342.3	48.8	17
MCH Service (Total Visits)	1425	1456.3	31.3	2

In order to determine whether there was a significant difference between the mean visits for each service and the overall number of service visits, we also performed a one-way ANOVA. As a result, there was a significant variation in the number of service visits during the comparison periods in the utilization of antenatal care services [*p* = 0.043, f (1.22) = 4.60] (Table 5).

**TABLE 5:** One-way analysis of variance for antenatal care by period of service utilization during COVID-19 at selected health facilities (Eastern Ethiopia, 2021).

Source	df	SS	MS	f	p
Between groups	1	14308.17	14308.17	4.601	0.043
Within groups	22	68421.67	3110.08		
Total	23	82729.83			

## Discussion

In Ethiopia, during the time of the Covid-19 pandemic, we observed a disruption in the delivery of MCH services in the study region. Similarly, there is evidence demonstrating that outbreaks have impacted the social, economic, and health systems. COVID-19 crippled the healthcare system and created a serious crisis [5, 6]. Evidence from the WHO study, Global Financing, and other studies confirmed that the existence of COVID-19 had severely disrupted most health services, including essential health services like MCH [7–14]. The adoption of a range of inconsistent policies and interventions during the COVID-19 pandemic may have contributed to the disruption of MCH service. In Tanzania, more emphasis was placed on COVID-19 measures and less on primary healthcare. Among other things, a major contributing factor in the disruption of MCH services was the shortage of essential commodities, vaccines, and medical supplies [6]. Furthermore, disruptions may have come from heightened fear of receiving COVID-19 or being sent to an isolation center, staff shortages brought on by structural adjustments and reassignments in response to the pandemic, postponed visits and reduced access to transportation options [6, 15, 16].The utilization of MCH services was restricted due to the pre-existing weak health system, limited capacity for healthcare, reallocation of resources, and financial constraints [28]. In addition, fear of the pandemic caused by societal and mainstream media preconceptions, in addition to the statewide implications of preventive measures, limited public mobility to health facilities. As a result, the health of mothers and children may have been adversely affected in the final months of the pandemic [29].

Since the presence of the COVID-19 virus was confirmed in Ethiopia, postnatal care service utilization has drastically decreased. Similarly, studies conducted in South Africa, Ethiopia, and Rwanda found that postnatal care services significantly decreased in the months that followed the COVID-19 announcement [6, 10, 11, 17]. Despite the federal Ministry of Health’s advocacy for PNC, mothers and medical professionals often refused to use PNC services. In addition, lack of personal protective equipment (PPE) and medical supplies, changes in routine services due to structural adjustments, and fear of contracting COVID-19 may have all led to a decline in service use [6, 15]. Consequently, there may be evident morbidities in mothers and newborns immediately after delivery [30]. Postpartum maternal morbidity, delayed initiation of child immunization programs, and fewer opportunities to identify high-risk newborns further complicate maternal and child health.

In the 3 months following Ethiopia’s declaration of a COVID-19 pandemic, there was a decrease in the use of family planning services in the study sites. A study carried out in the northern Ethiopian Tigray region also confirmed a 4.81% decrease in family planning services during the COVID-19 period [15]. Similar declines have been reported in family planning services in Bangladesh, South Africa, Nigeria [10], Mozambique [18], Dessie [26], and the southwestern regions [20] of Ethiopia. Low utilization of family planning services in Tanzania has been attributed to structural changes, such as changes in clinic scheduling and staff availability, problems in obtaining and distributing medical supplies, and fear of contracting COVID-19 while using family planning services [25]. However, a study in Kenya found that adolescents were using injectables and short-term family planning more frequently during that period [15]. In Tanzania, soon after the pandemic, new clients began to use family planning services again [25]. Numerous studies have documented various types of violence, sexual abuse, and assault that occurred during the COVID-19 pandemic, which may have created a need for family planning services [16, 18–22]. These may have resulted from travel restrictions and school closures, which made family planning services more necessary in the later stages of the COVID-19 pandemic. Reduced use of family planning services in the months following the declaration of a COVID-19 pandemic in Ethiopia could place women and girls at risk for unplanned pregnancies, unsafe abortions, and associated morbidity and severe consequences to the mother [36, 37].

Around the time of the COVID-19 declaration, delivery services at the study facilities were reduced for approximately 4 months. Results from global studies, in addition to those from Ethiopia, Asia, and Sub-Saharan Africa, strongly support this conclusion. In those cases, the studies found a notable decline in facility deliveries during the COVID-19 pandemic [8, 10, 12, 17, 23–27]. Pregnant women’s perceptions of receiving less care than expected because of various constraints and disruptions in the health system could constitute the cause of the decline in facility deliveries. Differences in the sources of data and the study period considered may be another factor [15]. In addition, pregnant women’s fear of contracting COVID-19 combined with unavailability and inadequate services, and lack of postpartum beds and rooms to ensure physical distance, may have forced mothers to at-home deliveries, cesarean sections, and referrals to higher tier levels [25]. Complicated pregnancy outcomes from home deliveries have the potential to affect the health of the mother and the newborn. Referrals and Cesarean sections may have made it more difficult for families to pay for care, increasing the financial impact of the pandemic.

One month after the COVID-19 existence was announced in Ethiopia, this study found an abrupt drop in child vaccination services. In a similar vein, compared to comparable times the previous year, there was a decline in the use of child vaccination services. The decrease in child immunization services since the COVID-19 announcement has also been documented by several studies, including one that was conducted globally in 63,000 health facilities [8], one that was conducted multi-nationwide in Bangladesh, Nigeria, and South Africa [10], India [13], Sub-Saharan Africa [14], Rwanda [16], Mozambique [18], and one in Ethiopia [15, 26]. Low child immunization service utilization in Tanzania has been linked to a number of issues, such as disruptions in the procurement and distribution of medical supplies, shortages of vaccines and related supplies, and rescheduling of clinic services [25]. As a result, these factors could lead to delayed initiation of vaccination, increased dropout rates, and an increased risk of vaccine-preventable infections in the later stages of the pandemic.

The results of the current study demonstrated that although the utilization of antenatal care services decreased in Ethiopia after the declaration of COVID-19, there was a modest increase in the later months. The findings are in line with research conducted in Ethiopia, Nepal, and Kenya, where ANC services declined in the early stages of COVID-19 [15, 24, 28]. Nonetheless, other studies demonstrated that the presence of COVID-19 had a significant negative influence on ANC [14, 16, 19–21]. This impact may have been caused by several factors, such as the absence of guidelines for ANC during the pandemic, fear of contracting COVID-19, long waiting times for ANC, hesitancy to get vaccinated against COVID-19, staff reallocations, rescheduling of appointments, a lack of medical supplies and PPE for basic health services, and persistent hesitation to use the services [14, 25]. This could potentially challenge the 2016 WHO recommendation that ANC visits should be increased to eight during the time of each pregnancy [29], as there may be a greater chance of missing opportunities to detect high-risk pregnancies early on, which is essential to preventing maternal mortality.

In summary, we found that the utilization of maternal and child health services was impacted in a variety of ways by COVID-19. The majority of MCH service utilization, both before and after the COVID-19 outbreak was announced in Ethiopia, declined in close proximity to that date. There were statistically significant decreases in antenatal care, delivery, and child immunization services. However, a few months after Ethiopia’s COVID-19 announcement, mothers resumed seeking services.

Perhaps the Federal Ministry of Health of Ethiopia’s (FMoHE) establishment of a novel emergency response framework specifically created for the prevention and management of COVID-19 has stabilized the service. A family outreach team, consisting of a nurse, midwife, laboratory technician, health extension worker, and local guide, has been formed by the FMoHE. The family team checks every home in its specified catchment area, paying special attention to expectant mothers and young children under the age of five. The group offers COVID-19 screening, health education, and any other emergency medical assistance. Mothers are then encouraged to use the facilities’ services, while local guides and health extension workers handle follow-up visits.

### Limitations and Strengths of the Study

The primary source of data for the current study was facility records, and the accuracy and completeness of the data could have an impact on the findings of the study. In addition, due to data availability, we were not able to analyze individual-level characteristics, such as sociodemographic or economic factors. Furthermore, seasonal factors should be taken into account when discussing MCH service utilization [30–35].

One of our strengths was that we incorporated a range of maternal and child health services, and a full year of service utilization patterns to comprehend the effects of COVID-19 in a broader context.

### Conclusion and Recommendations

The findings of this study suggest that the COVID-19 pandemic had a substantial impact on maternal and child healthcare. Notably, several services have experienced a visible decline, including family planning, institutional delivery, and child immunization services. This suggests that a sizable proportion of mothers who should have attended ANC, expected to give birth in a facility, and used PNC services may have missed the service. There was also a decline in the utilization of family planning services, which increases the likelihood of unwanted or unplanned pregnancies, and subsequently the risk of abortion. The findings further implied that there were children who had not started their vaccinations at the right age, children who had dropped out of the routine vaccination schedule, and a higher possibility of outbreaks of vaccine-preventable diseases.

In order to reach children who have missed or stopped receiving their vaccinations, mothers who recently gave birth and did not visit the facility for PNC or FP services, and pregnant mothers who are in need of ANC services, health system managers should strengthen and mobilize the workforce at the lower service delivery inlets, health posts, and focus their approach on community outreach services. Simultaneously, customized communication approaches should be put in place in order to allay and counterbalance the fear and anxiety caused by the pandemic.

Ultimately, policymakers and healthcare managers should endeavor to strengthen the resilience of the health system to deal with pandemics and local epidemics, and develop novel strategies to enable the healthcare system to act efficiently and reliably.

## Abbreviations

ANC, Antenatal Care; ANOVA, Analysis of Variance; DDU, Dire Dawa University; ED, Emergency Department; EDHS, Ethiopian Demographic Health Survey; EMDHS, Ethiopian Mini Demographic Health Survey; ETB, Ethiopian Birr; FMoH, Federal Ministry of Health; JSI, John Snow International; KM, Kilo Meter; L10K, Last 10 Kilo Meter; MCH, Maternal and Child Health; MNCH, Maternal Neonatal and Child Health; PNC, Post Natal Care; RERC, Research Ethics Review Committee; RMNCAH, Reproductive, Maternal, Newborn, Child and Adolescent Health; SARS, Severe Acute Respiratory Syndrome; SPSS, Statistical Package for Social Sciences; WHO, World Health Organization.
